# Addition of Selected Plant-Derived Semiochemicals to Yellow Sticky Traps Does Not Improve Citrus Psyllid Captures

**DOI:** 10.1007/s10886-024-01491-0

**Published:** 2024-04-03

**Authors:** Dylan A. Pullock, Kerstin Krüger, Aruna Manrakhan, Abdullahi A. Yusuf, Christopher W. Weldon

**Affiliations:** 1 Department of Zoology and Entomology, Forestry and Agricultural Biotechnology Institute (FABI), University of Pretoria, Hatfield, Pretoria, 0028 South Africa; 2 KWS SAAT SE & Co. KGaA, Einbeck, 37574 Germany; 3 Citrus Research International, Mbombela, 1200 South Africa; 4 Department of Conservation Ecology and Entomology, Faculty of AgriSciences, Stellenbosch University, Stellenbosch University, Private Bag X1, Stellenbosch, Matieland, 7602 South Africa

**Keywords:** *Diaphorina citri*, *Trioza erytreae*, Odorants, Huanglongbing, Temperature, Integrated pest management

## Abstract

Fast and effective monitoring and surveillance techniques are crucial for the swift implementation of control methods to prevent the spread of Huanglongbing, a devastating citrus disease, and its invasive psyllid vector, Asian citrus psyllid, *Diaphorina citri*, into South Africa, as well as to control the native vector, African citrus triozid, *Trioza erytreae*. Monitoring for citrus psyllid pests can be improved by using semiochemical odorants to augment already visually attractive yellow sticky traps. However, environmental variables such as temperature and humidity could influence odorant release rates. Five field cages were used to test the ability of a selection of odorants to improve yellow sticky trap efficacy in capturing citrus psyllids. Environmental effects on odorant loss from the dispensers were also investigated. The odorants that most improved yellow sticky trap captures in field cages were then tested under open field conditions alongside lower concentrations of those same lures. Gas chromatography-mass spectrometry was used to calculate odorant release rates as well as to determine if any contamination occurred under field conditions. None of the odorants under field cage or field conditions significantly improved psyllid capture on yellow sticky traps. Temperature influenced odorant loss, and release rate from polyethylene bulbs decreased over time. Based on these results, the use of unbaited yellow sticky traps seems to be the most effective method for monitoring of Huanglongbing vectors.

**Supplementary Information:**

The online version contains supplementary material available at 10.1007/s10886-024-01491-0.

## Introduction

Surveillance is important for the early detection of new and potentially invasive species, some of which may be of health or phytosanitary concern. Due to this and the strong economic factors for doing so, the development of effective and efficient monitoring techniques is a major focus of research on invasive pest species (Augustin et al. 2012; Brockerhoff et al. 2013; Epanchin-Niell et al. 2014; Ilyas et al. 2017; Moir et al. 2022; Poland and Rassati 2019). For example, France experienced a ‘damage-loss’ cost of US$732 million due to biological invasions between 2009 and 2012, and a cumulative cost of US$258 million to the French agricultural sector during the period of 1993 to 2018 (Renault et al. 2021). Likewise, Brazil lost approximately US$104.33 billion as a result of damage caused by invasive alien species over a period of 35 years (1984–2019), 40% of which were incurred in the agricultural sector (Pires Adelino et al. 2021). Diagne et al. (2021) have also estimated that the continent of Africa has accumulated an invasive alien species-related cost of US$78.9 billion from 1970 to 2020, of which US$34.2 billion was attributed to the agricultural sector. To avoid these costs, effective surveillance can aid in the speedy and economic implementation of suitable control methods to curb the establishment, spread and damage to crop production or native ecosystems caused by invasive alien species (Epanchin-Niell et al. 2014; Epanchin-Niell and Liebhold 2015).

Citrus infesting psyllids, as either invasive alien or native species, are economically significant pests in countries with a citrus industry. For example, the Asian citrus psyllid, *Diaphorina citri* (Kuwayama) (Hemiptera: Psyllidae), is an invasive alien species present in some of the major citrus producing regions of the USA (California, Florida, and Texas), Mexico (Michoacán, Veracruz, and Tamaulipas), Brazil (São Paulo and Paraná), Tanzania (Morogoro), and China (Guangdong, Guangxi, Fujian, Zhejiang, Hunan, Yunnan, and Sichuan) (Bassanezi et al. 2020; Gonzalez Cardenas et al. 2012; Graham et al. 2020; Luna-Cruz et al. 2018; Shimwela et al. 2016; Wang et al. 2022; Yang et al. 2006; Zavala-Zapata et al. 2022). Likewise, the African citrus triozid, *Trioza erytreae* (Del Guercio) (Hemiptera: Triozidae), is an invasive alien species present in the Iberian peninsula whose spread threatens important citrus growing regions within southern Portugal and Spain (Arenas-Arenas et al. 2019; Ruíz-Rivero et al. 2021). Similarly, both *D. citri* and *T. erytreae* are important pests in countries that make up a part of their native distribution. *Diaphorina citri*, believed to originate from the Indian subcontinent before spreading to the rest of Asia (Hall 2008; Hollis 1987), causes significant damage to acid lime in northern Karnataka, which is one of India’s important citrus growing regions (Aruna and Jagginavar 2017). As for *T. erytreae*, a psyllid species with possible origins in southern Africa (Ajene et al. 2020a; Lounsbury 1896; Van Den Berg 1990), South Africa has successfully implemented control strategies for it, and the African citrus greening (ACG) disease that it is a vector for, since the 1980’s (Roux and Buitendag 1896). It is important to note that *T. ertyreae* was considered a major citrus pest in South Africa before these control methods were developed (McClean and Oberholzer 1965), and routine monitoring as well as control of the species is ongoing due to it still being considered a pest of economic significance (Grout 2022). Monitoring involves inspecting the edges and underside of immature leaves for the presence of one or more *T. erytreae* eggs or nymphs (Grout 2022). Due to a zero-tolerance treatment threshold, control options are implemented if even one egg or nymph is found (Grout 2022). These control methods include soil and foliar spray treatments with chemical plant protection products (Grout 2022).

Damage by citrus infesting psyllids can result in reduced plant photosynthetic capacity, induced by sooty mould growth assisted by the honeydew that psyllids produce, as well as die-off of new flush due to heavy feeding (Burckhardt 1994; Cocuzza et al. 2017; Rwomushana et al. 2017). However, the direct damage of both *D. citri* and *T. erytreae* on citrus is of less concern than their status as vectors for the Asian citrus greening disease, Huanglongbing (HLB and, to a lesser extent, ACG (Roux and Buitendag 2003). HLB is one of the most damaging citrus diseases globally (Bove 2006). It is present on the North American, South American, Asian, and African continents (Bassanezi et al. 2020; Graham et al. 2020; Shimwela et al. 2016; Tipu et al. 2017). On the African continent, HLB is present in Ethiopia and Kenya, having first been detected in Ethiopia in 2009 and then in Kenya around 2017/2018 (Ajene et al. 2020c; Saponari et al. 2010). In African countries where HLB is not yet present but its psyllid vectors are found, there is a risk of HLB spreading to citrus production zones (Aidoo et al. 2023; Ajene et al. 2020b, c; Oke et al. 2020; Rwomushana et al. 2017; Saponari et al. 2010; Sétamou et al. 2023; Shimwela et al. 2016). Huanglongbing, like ACG, is associated with a gram-negative, ɑ-Proteobacteria ‘*Candidatus* Liberibacter’ group. African citrus greening is associated with ‘*Candidatus* Liberibacter africanus’ (CLaf) while HLB is associated with ‘*Candidatus* Liberibacter asiaticus’ (CLas) (Damsteegt et al. 2010; Jagoueix et al. 1994; Yu and Killiny 2020). Both CLas and CLaf are restricted to the phloem within plants (George et al. 2019; Graca 1991; Roberts et al. 2021). Huanglongbing causes shoot yellowing as well as fruit malformation, premature fruit drop, and tree dieback, all affecting fruit marketability and production, with infected citrus trees persisting for several unproductive years before death (Aidoo et al. 2020; Bove 2006; Saponari et al. 2010). When psyllid vectors feed from the phloem they become infected with the causative bacteria and transmit it to other trees when feeding. As such, monitoring and the control of psyllid vectors are important measures to prevent the spread of both the vectors and HLB.

Host finding by psyllids, where host plants are those in which psyllids complete their life cycle (Burckhardt et al. 2014), is a complex process that makes use of visual, olfactory, and gustatory cues (Benhadi-Marin et al. 2021a; Meng et al. 2022; Patt and Sétamou 2010; Sétamou et al. 2014). Yellow sticky traps are used for *D. citri* and *T. erytreae* collection at low population densities, relying heavily on their visual attractiveness to make them a suitable monitoring tool (Benhadi-Marin et al. 2021b; Miranda et al. 2018; Monzo et al. 2015). However, this effectiveness can be influenced by variation in population sizes, particularly for *D. citri* (Amoros et al. 2019). The use of semiochemicals in conjunction with yellow sticky traps can improve the effectiveness of these traps for monitoring of citrus psyllids like *D. citri* (Amoros et al. 2019; Coutinho-Abreu et al. 2014; Patt and Sétamou 2010; Sétamou et al. 2014). Plant-derived semiochemicals also show promise for improving captures of *T. erytreae* on yellow sticky traps (Antwi-Agyakwa et al. 2019; Benhadi-Marin et al. 2021a, b). These same studies show a possible overlap in some of the semiochemicals either individually or as part of a blend that *D. citri* and *T. erytreae* find attractive, meaning it may be possible to improve yellow sticky trap efficacy for both HLB vector species simultaneously, and thereby increase the sensitivity of surveillance for both at the same time (Antwi-Agyakwa et al. 2019; Coutinho-Abreu et al. 2014; George et al. 2019; Lapointe et al. 2016; Mann et al. 2012; Patt and Sétamou 2010; Zanardi et al. 2019). There is also the possibility of psyllid response to semiochemicals being associated with their rate of release (Martini et al. 2016, 2020). However, this is rather complex because the release rate of semiochemicals might be a function of temperature, which could result in an increase or decrease in psyllid response depending on temperature. In fact, Bradley et al. (1995) developed a model for predicting pheromone release rates from polyethylene tubing dispensers that was reliant on temperature. In addition, humidity may influence dispersal of odorants from a dispenser (Tomaszewska et al. 2005).

In this study, we aimed to improve the efficacy of yellow sticky traps as a surveillance tool for HLB psyllid vectors using South Africa’s native HLB vector psyllid species, *T. erytreae*, as the study species. This was done by testing ten semiochemicals (one commercially available *D. citri* lure blend, eight single plant volatile compounds that *D. citri, T. erytreae* or both have responded to in laboratory, semi-field or field trials) under field cage conditions. The most effective semiochemicals from the field cage choice tests were then tested under open field conditions. Release rates of each chemical were also quantified, which we predicted would increase with temperature. The use of semiochemical lures was expected to improve trap performance for *T. erytreae* by combining visual and chemical cues with attractive qualities, which would increase psyllid captures on traps and improve their sensitivity.

## Methods and Materials

### Psyllid Handling and Maintenance

The citrus psyllids used in this study were sourced from an existing culture held on the Hatfield campus of the University of Pretoria, South Africa. The culture was established in 2021 with it being frequently refreshed when wild nymphs were found. This culture was established using *T. erytreae* nymphs collected from lemon [*Citrus limon* (L.) Osbeck] and white ironwood [*Vepris lanceolata* (Lam.) G. Don] trees found around the suburbs of Johannesburg and Pretoria, South Africa. The culture was housed in custom made wooden cages with a top and front glass panel and gauze at the sides for ventilation (46 cm × 46 cm × 92 cm) with potted, flushing white ironwood plants. White ironwood is an alternative host on which *T. erytreae* oviposit and nymphs can complete development (Moran 1968; Roberts et al. 2015). The *T. erytreae* culture was kept in a climate room at 23 °C ± 1 and a relative humidity of 60–80%, with a 14:10 h light: dark cycle with dusk and dawn simulated where the lights turn on at 06:00 and off at 20:00 (South Africa Standard time; GMT + 2). The rearing temperature resulted in one generation lasting roughly a month, but there was overlap in generations due to refreshing the culture. The lights used were a 1:1 mix of fluorescent cool white and plant growth light tubes to ensure proper lighting and health for each plant used to rear the culture. When introducing wild-collected nymphs to the culture, they were first stored separately in small pill containers until the adults emerged. This prevented the introduction of psyllid parasitoids into the psyllid culture cages and the identity of the psyllids could be visually confirmed based on morphology (EPPO 2005). These newly emerged psyllids were introduced to the culture cages so that they could propagate on the fresh white ironwood flush.

A separate cold room, set at a temperature of 16 °C with the other parameters being the same as the rearing room, was used to induce flushing in several ironwood plants in rotation. This ensured that there were always flushing white ironwood plants for use as a food source and oviposition substrate by the psyllid culture. These ironwood plants were kept in the cold room for a week before being introduced to the rearing room and left until the new flush developed before psyllids from the old cage were introduced to new plants housed in a different cage. By doing so we could maintain and increase the number of psyllids for use in the field cage trials.

### Chemicals

The semiochemicals used in this study were acetic acid (≥ 99%; A6283), (*R*)*-*(+)-limonene (97%; 183,164), sabinene (75%; PHL82342), an ocimene isomer mix comprising cis-ocimene and ß-ocimene of (≥ 90%; W353901), myrcene (≥ 90%; 64,643), ethyl butyrate (99%; E15701), methyl salicylate (≥ 99%; M6752), p-cymene (99%; C121452), and hexane (≥ 99%; 32,293) as the nontoxic organic solvent. All semiochemicals were obtained from Sigma Aldrich. Eight of the chemicals have been reported to elicit a response in *D. citri* and/or *T. erytreae* under laboratory, semi-field or field conditions and may be attractive in nature (Antwi-Agyakwa et al. 2019; Coutinho-Abreu et al. 2014; Zanardi et al. 2019). Hexane was the organic nontoxic solvent used for all test chemicals. Additionally, a commercially available *D. citri* lure called ACP Pherolure (Insect Science, South Africa) was tested. ACP Pherolure is a blend of ɑ-phellandrene, ß-phellandrene, ß-caryophyllene and methyl salicylate, which could potentially attract *T. erytreae*. The eight single chemicals selected from the literature were used undiluted from their respective bottles to test if highly concentrated chemicals could improve attractiveness of yellow sticky traps in an initial screening to identify promising chemicals for further studies. A sample of 4000 µL of each chemical was transferred to a polyethylene bulb, which was then heat sealed using a hot metal spatula.

### Field Cage Trial

Ten chemicals listed above were tested in field cage trials under choice conditions. The field cage choice tests were run using five custom made field cages (160 × 160 × 180 cm) that were set up at least 20 m apart from any other field cage on an open plot of land on the University of Pretoria’s Innovation Africa campus (S 25.75071; E 28.26017) in Koedoespoort 456-Jr, Pretoria (Fig. S1). This 20 m buffer was to prevent possible overlap of the different semiochemicals tested in the five cages. Within each of these cages, two potted lemon trees were placed diagonally opposite one another and at least 50 cm apart. Two yellow sticky traps (14 cm × 20 cm; Insect Science, South Africa), one baited with a sealed polyethylene bulb dispenser containing one of the 10 chemicals and one unbaited with just an empty polyethylene bulb, were hung with one placed in each tree while maintaining at least a 50 cm distance between the traps. An iButton (1-wire Hygrochron, Analogue Devices Incorporated, Wilmington, USA) was included in one of the field cages to record temperature and humidity every 10 min for the full week period. After the traps were hung, 20 two-week-old *T. erytreae* psyllids (10 males and 10 females) were collected from the culture using an aspirator, transferred to vials and released into each field cage. This age group was selected because it is most responsive to plant volatiles (Antwi-Agyakwa et al. 2019). The yellow sticky traps were inspected at 10 min, 3, and 7 days after release and the catches were recorded for data analysis. The 10-minute observation was included to assess if the semiochemical in the bulb would lead to an immediate and obvious response relative to the accompanying unbaited blank yellow sticky trap. After 7 days, the traps were wrapped in plastic kitchen film (330 mm × 50 m, Supermama, South Africa) and stored in a refrigerator to prevent specimen deterioration. The weight of each odour filled bulb was determined at days 0, 3, and 7 so that chemical loss could be calculated and correlated with psyllid catch. Nine replicates were run for each semiochemical between 27 May 2022 and 6 September 2023.

### Open Field Trial

The two best performing semiochemicals from the field cage choice tests were the ocimene isomer mix and ethyl butyrate. These two semiochemicals were selected for further testing under open field conditions because they were the only ones that achieved a higher (although not significantly so) psyllid catch than the unbaited trap. Alongside them, a 1% ocimene mixture and a 5% ethyl butyrate mixture (both diluted with hexane), as well as hexane control were also included in the field trial. These diluted concentrations were selected based on a study by Coutinho-Abreu et al. (2014) and were included to see if the low chemical concentrations that elicited a response in *D. citri* under lab conditions would lead to a similar response in *T. erytreae*. The different chemicals were pipetted into polyethylene bulbs at the same volume as in the field cage trials and sealed. These, as well as a control comprising only empty, sealed polyethylene tubes, were attached to yellow sticky traps using a piece of wire.

The open field trial was run in a pesticide free experimental lemon orchard, where *T. erytreae* occurs naturally, at the University of Pretoria’s Innovation Africa campus in Hillcrest. Extensive past and recent damage on the leaves of the lemon trees and preliminary placement of yellow sticky traps confirmed the presence of adult *T. erytreae* in the orchard. Each polyethylene bulb was weighed at Days 0, 3, and 7 to record loss of the chemical contents. Each trap was placed roughly 1.5 m above the ground in the outer canopy of their own tree in a 5 × 6 grid pattern with a distance of 4 ± 1 m between traps. The position of the traps in the grid were randomised for each tested week (Table S1). The exceptions in distance were caused by missing trees in the orchard, resulting in distances in some rows being 7–10 m between trees. An iButton was also hung up in the outer canopy of a tree in the centre of the orchard testing area to record temperature and relative humidity every 10 min for the 7-day period of each test. After 7 days, the traps were collected and wrapped with plastic kitchen film for transport and storage. The traps were inspected for psyllid catch at 10 min, 3, and 7 days with the numbers caught being recorded cumulatively. Traps checked after 10 min and 3 days were done manually in the field, with the 7-day check being done in the lab under a microscope (Model SZ61, Olympus corporation, Tokyo, Japan) after the collection, wrapping and transport, but before storage. This was repeated for another four weeks, totalling 5 weeks over a period from 30 June 2023 to 4 September 2023, with 5 traps per treatment per week.

During the field trial, we evaluated the composition and concentration of volatiles being released from the polyethylene bulbs. This was to determine if any contamination of the odorant was occurring in the field or if there was any formation of secondary compounds under field conditions. Five bulbs, each one containing one of the five semiochemical treatments, were taken for volatile entrainment before being deployed in the field as part of the field trial. They were then attached to their relevant traps and deployed in the field, where they were allowed to age in the orchard before volatiles were entrained again at 3 and 7 days. This was repeated for each of the five weeks. A bag blank as well as a bag-and-bulb blank were included to account for volatiles released from the bags and the polyethylene bulbs.

### Volatile Collection

Volatiles from the bulbs containing the chemicals were trapped using a push-pull system as described in Schröder et al. (2015). Briefly, this entails pushing charcoal scrubbed air at a flow rate of 1 L/min split between three oven bags (Pick n Pay Medium Roasting bags, 250 mm × 400 mm) and pulled at 0.33 L/min into HayeSepQ traps for one hour. The bags were fully inflated by the push pump before cutting off a corner. The push pump delivering the charcoal-filtered air into the system was allowed to run for a further hour before the experiments started to flush out any tainted air from the bags. Afterwards, the HayeSepQ adsorbent traps were inserted into the open corners of the bags and sealed in place using polytetrafluoroethylene (PTFE) tape. Thereafter, the traps were removed, eluted using 500 µL of dichloromethane (DCM) under a gentle stream of nitrogen, and the extract was stored at -80 °C prior to analysis. This was repeated over an additional 4 weeks, resulting in five replicates of each odorant at fresh, day three, and day seven as well as five replicates of the bag only, and bag and bulb controls corresponding to each week. The entrainment equipment was set up and entrainments were done in a clean, dust-free room at a controlled temperature of 25 °C.

### Chemical Analyses and Volatile Release Rates

One µL of each extract was injected at 250 °C in the split mode (split ratio 1:10) onto a Shimadzu Q2100 SE gas chromatograph-mass spectrometer (GCMS) (Shimadzu Corporation, Japan) equipped with a Rtx5-MS (5% phenyl- methylpolysiloxane) GC capillary column (30 m × 0.25 mm ID × 0.25 μm film thickness) (Restek Corporation, Bellefonte, PA, USA) and helium as the carrier gas at a flow rate of 1.0 mL/min. The oven temperature was programmed at 50 °C for three minutes, increased at the rate of 20 °C/min to 150 °C, and ramped at 10 °C/min to 250 °C. The mass selective detector was set at 230 °C, with an ion source temperature of 200 °C, and electron energy of 70 eV was used to obtain electron impact (EI) mass spectra while the fragment ions were analysed over the mass range of 50–700 m/z in the scan mode with a scan speed of 2500 per 0.30 s. The qualitative identification of compounds was done by comparing the mass spectrometric data and retention times to those of reference spectra published in the NIST 08, 11 and Wiley 08–MS libraries and confirmed by synthetic standards (ocimene isomer mix ≥ 90% and ethyl butyrate 99% purity, Sigma Aldrich) (Fig. S2). The release rates of the volatiles were quantified using linear calibration curves obtained from chromatograms of a serial dilution (1 × 10^− 6^ – 10%) of the authentic standard ocimene isomers and ethyl butyrate that were analysed using the same GC-MS method as those of the volatiles. The linear equations generated were $y=5{e}^{9}x+2{e}^{7}$; R^2^ = 0.9986 for ocimene isomers and $y=2{e}^{10}x-2{e}^{6}$ ; R² = 0.9973 for ethyl butyrate. Using the density provided on the solvent bottles, the average volume of the odorants in the bulbs used for entrainment were calculated using their recorded weights for Days 0, 3, and 7 to compare the average volume of odorant in the entrained bulbs to the average % release rate of the volatile semiochemicals.

### Statistical Analysis

Data analysis was done using R version 4.3.1 (R Core Team 2021) and RStudio version 2023.6.0 (RStudio Team 2021). To determine if there was a significant difference in psyllid catch between baited and unbaited traps, Wilcoxon signed-rank tests were run to compare psyllid numbers caught on baited yellow sticky traps relative to their unbaited counterparts in the same cages after 3 and 7 days. Kruskal-Wallis tests were also run between the semiochemicals at days 3 and 7 to determine if any of the semiochemicals performed better than the others. Linear regression models were run for each odorant, with a model minimisation step to obtain the minimum adequate models for each odorant, to determine the effect of temperature as well as humidity on odorant loss after 3 days. The day 3 data were used because hexane was still present in the bulbs after 3 days in the field but not after 7 days. A response surface for each semiochemical was generated to visualise the effect of temperature and humidity on semiochemical loss. For the field trials, a generalised linear model (GLZ) with a negative binomial distribution was used to determine the effect of treatment and day on psyllid catch. The odTest function in the pscl R package (Jackman 2017) identified that a negative binomial distribution was a better fit for the data than a Poisson distribution due to overdistribution of the trap capture data. Then likelihood ratio tests for the negative binomial model were run using the Anova function (car package) to confirm the effects of treatment and day on psyllid catch.

## Results

### Catches of Psyllids Using Baited and Unbaited Traps in Field Cages

**Fig. 1:**
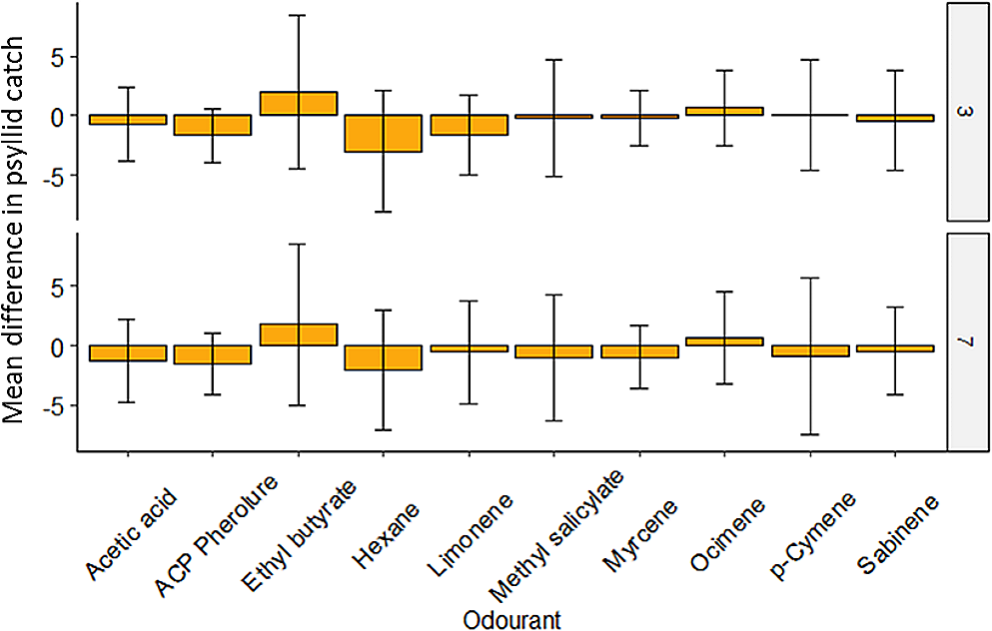
The mean difference in psyllid catch between baited traps relative to their unbaited counterparts after 3 and 7 days in the field cages. Positive values indicate that more psyllids on average were caught on baited traps whereas negative values indicate that on average unbaited traps caught more psyllids. Error bars show ± 1 standard error of the mean

No semiochemical tested in the field cages significantly improved psyllid catch in comparison with unbaited yellow sticky traps after 3 or 7 days (Table S2; Fig. S3). Furthermore, comparing psyllid catch between the different odorants indicated that no odorant led to significantly higher psyllid catches on yellow sticky traps after 3 (Kruskal-Wallis test, *H* = 8.16, df = 9, *P* = 0.518) and 7 (*H* = 4.73, df = 9, *P* = 0.857) days. Ethyl butyrate and the ocimene isomer mix were the only tested semiochemicals that had an average positive psyllid catch, indicating that these baited traps caught more psyllids that their unbaited counterparts on average (Fig. 1).

### Effects of Environmental Variables on loss of Semiochemicals

Temperature played a significant role in the weight loss of semiochemicals from polyethylene bulbs deployed in field cages, with release increasing as temperature increased (Fig. 1; Table 1). Out of all the tested semiochemicals, only ethyl butyrate was significantly affected by humidity and its interaction with temperature (Table 1).

**Table 1:** Effects of temperature and humidity on odorant loss over a three day period. In each case, the minimal adequate model is presented

Odorant	Estimate	Std error	t	Pr (>|t|)	Odorant	Estimate	Std error	T	Pr (>|t|)
**Acetic acid**					**Limonene**				
(Intercept)	0.794	11.714	0.068	0.949	(Intercept)	-3734.225	1715.115	-2.177	0.081
Temperature	1.147	0.420	2.730	**0.041**	Temperature	291.213	87.577	3.325	**0.021**
Humidity	-0.010	0.095	-1.054	0.340	Humidity	42.216	25.460	1.658	0.158
**ACP Pherolure**					Temperature × Humidity	-3.006	1.422	-2.115	0.088
(Intercept)	-44.508	82.552	-0.539	0.613	**Methyl salicylate**				
Temperature	25.083	2.526	9.931	**< 0.001**	(Intercept)	-14.450	15.281	-0.949	0.386
Humidity	1.339	1.128	1.187	0.289	Temperature	3.253	0.592	5.491	**0.003**
**p-Cymene**					Humidity	-0.202	0.117	-1.738	0.143
(Intercept)	-275.61	107.04	-2.575	**0.037**	**Myrcene**				
Temperature	37.25	6.08	6.126	**< 0.001**	(Intercept)	-108.820	100.183	-1.086	0.319
**Ethyl butyrate**					Temperature	25.013	3.617	6.916	**< 0.001**
(Intercept)	-672.290	218.922	0.068	**0.028**	Humidity	-1.090	0.864	-1.262	0.254
Temperature	44.612	10.894	2.730	**0.009**	**Ocimene**				
Humidity	10.643	3.454	3.081	**0.027**	(Intercept)	-330.848	85.077	-3.889	**0.006**
Temperature × Humidity	-0.602	0.176	-3.430	**0.019**	Temperature	33.445	5.095	6.564	**< 0.001**
**Hexane**					**Sabinene**				
(Intercept)	-770.242	329.398	-2.338	0.058	(Intercept)	-1411.070	742.207	-1.901	0.116
Temperature	112.765	10.041	11.231	**< 0.001**	Temperature	95.299	33.713	2.827	**0.037**
Humidity	4.837	3.980	1.215	0.270	Humidity	16.920	10.646	1.589	0.173
					Temperature × Humidity	-0.996	0.503	-1.982	0.104

**Fig. 2:**
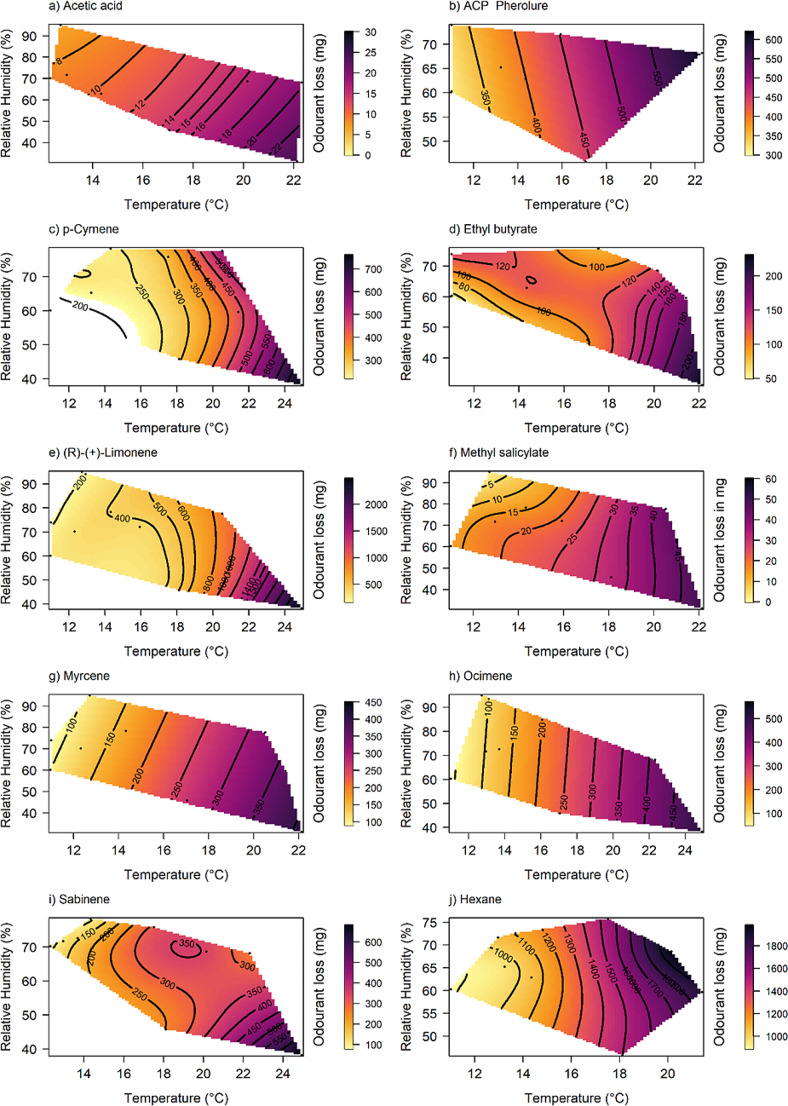
Response surfaces depicting the effect of temperature and relative humidity on odorant loss for the ten tested semiochemicals over 3 days: (**a**) Acetic acid, (**b**) ACP Pherolure, (**c**) p-Cymene, (**d**) Ethyl butyrate, (**e**) (*R*)-(+)-limonene, (**f**) Methyl salicylate, (**g**) Myrcene, (**h**) Ocimene, (**i**) Sabinene, and (**j**) *n*-Hexane

### Catches of Psyllids in the Field Using Selected Treatments

**Fig. 3:**
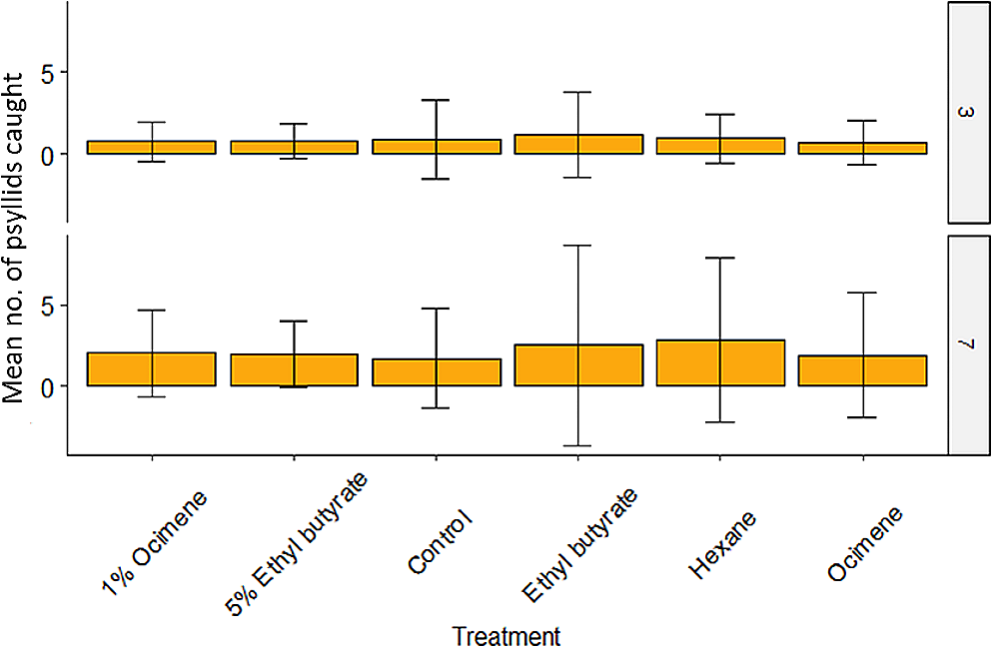
The mean number of psyllids caught after 3 and 7 days in the experimental lemon orchard by the different treatments. Error bars show ± 1 standard error of the mean

In the open field, treatment did not have a significant effect on psyllid catch (*LR χ*^2^ = 3.253, df = 5, *P* = 0.661). However, there was a significant difference between psyllid catch after 3 days and psyllid catch after 7 days (*LR χ*^2^ = 23.096, df = 1, *P* < 0.001). Ultimately, psyllid catch increased from day 3 to day 7 (Estimate = 0.916, Std. err = 0.185, *P* < 0.001; Fig. 3).

### Volatile Release Rates

The release rates of the standard volatiles followed a consistent pattern over the period of a week, with Day 0 having the highest and then decreasing through Day 3 and the lowest release rate being recorded at Day 7 as indicated by the difference in heights and area for all chemicals in the total ion chromatographs (Fig. S2). The average release rate for ethyl butyrate was 0.00468% per hour at Day 0 followed by 0.00345% per hour at Day 3 and 0.00125% per hour at Day 7. The average release rate for the 5% ethyl butyrate was smaller than their concentrated counterparts and recorded as 0.00331% per hour at Day 0, followed by 0.00208% per hour at Day 3, and 0.00064% per hour at Day 7. Previously, the isomer *β*-ocimene in the ocimene isomer mix was found to influence psyllid attractiveness (Amoros et al. 2019; Coutinho-Abreu et al. 2014). The average release rate for *β*-ocimene from the ocimene isomer mix at Day 0 was 0.01250% per hour, followed by 0.01091% per hour at Day 3, and 0.00538% per hour at Day 7. Like the situation between the average ethyl butyrate and 5% ethyl butyrate release rates, the *β*-ocimene release rate from the 1% mixture was smaller than the one from the undiluted odorant. They were recorded at 0.00030% per hour at Day 0, with 0.00023% per hour recorded at Day 3 followed by 0.00007% per hour at Day 7. This decrease in average release rate per hour from Day 3 to Day 7 coincided with an increase in odorant loss in mL from the dispensers during the same period. Weight losses equivalent to 0.0743 and 0.2423 mL of undiluted ethyl butyrate were recorded after 3 and 7 days respectively. For diluted ethyl butyrate solution (5% in hexane), weight losses equivalent to 0.0707 and 0.1334 mL were recorded after 3 and 7 days respectively. For undiluted ocimene, losses of 0.1613 and 0.3586 mL were calculated based on weight after 3 and 7 days. Diluted ocimene (1% in hexane) weight losses equivalent to 0.0141 and 0.0281 mL were recorded after 3 and 7 days.

## Discussion

Due to the literature indicating possible overlaps in the chemicals that both *D. citri* and *T. erytreae* find attractive, we predicted that at least some of the tested odorants would improve sticky trap efficacy. However, under both field cage and field conditions, none of the chemicals tested significantly improved the captures of *T. erytreae* by yellow sticky traps. This was unexpected as Coutinho-Abreu et al. (2014) found that odorants improved yellow sticky trap efficacy for *D. citri* by roughly 230% using a 2 mL loadout of diluted odorant (5% in paraffin oil) contained in a glass vial and then a plastic bag, with a bubble straw inserted to allow the odours to escape. The difference here could possibly be due to the difference in dispensers used (we used a sealed polyethylene bulb dispenser), which can influence the release rates of the volatiles they house thus impacting psyllid catch (Nielsen et al. 2019; Veršić Bratinčević et al. 2023). Furthermore, yellow sticky trapping has been shown to be effective at monitoring *T. erytreae*, and odorants (particularly limonene, sabinene, and *β*-ocimene) have been shown to be attractive to *T. erytreae* as well as *D. citri* (Antwi-Agyakwa et al. 2019; Benhadi-Marin et al. 2021b; Coutinho-Abreu et al. 2014). Other overlapping potential attractants include β-caryophyllene (Antwi-Agyakwa et al. 2019; Amoros et al. 2019) and myrcene (Antwi-Agyakwa et al. 2019; Coutinho-Abreu et al. 2014) which also added to that expectation. However, there are other possible explanations for the unexpected results of this study. Several studies that found that odorants were attractive to insects found that odorant blends were the most attractive, despite some studies noting that single chemicals were also attractive or elicited a response (Amoros et al. 2019; Antwi-Agyakwa et al. 2019; Coutinho-Abreu et al. 2014; Guarino et al. 2011; Khadka et al. 2020; Massa et al. 2008; Veršić Bratinčević et al. 2023). Khadka et al. (2020), in particular, found methyl salicylate to be more effective than semiochemical blends in improving catches of *D. citri* on sticky traps. Antwi-Agyakwa et al. (2019) and Amoros et al. (2019) also demonstrate how concentration or dosage of the odorant or odorant blend influence its attractiveness to target insects. The three odorant blend developed by Antwi-Agyakwa et al. (2019) lost its significant effect on *T. erytreae* at a higher dosage of 852 ng/µl compared to the effective 426 ng/µl dosage and Amoros et al. (2019) found that their attractive blend for *D. citri*, based on grapefruit volatiles, repelled the pest at a dosage just higher than the effective 0.1 mg loading dosage. A third possible explanation is that different isomers of the same odorant may be attractive to different citrus psyllids. Blends containing limonene were attractive to *D. citri* and *T. erytreae* (Amoros et al. 2019; Antwi-Agyakwa et al. 2019). However, Amoros et al. (2019) used (*R*)-(+)-limonene, or *D*-limonene, in a blend for *D. citri* whereas Antwi-Agyakwa et al. (2019) used (*L*)-(+)-limonene, or *S*-limonene, in two blends for *T. erytreae*. Our own study found that *D*-limonene did not significantly improve captures of *T. erytreae* on yellow sticky traps in the field, pointing to the possibility of a potential isomer effect.

The effect of temperature on chemical volatility seen in this study, where an increase in temperature resulted in an increase in odorant loss from sealed permeable dispensers, is common for volatiles (Bradley et al. 1995; Brown et al. 1992; Nielsen et al. 2019; Torr et al. 1997). As temperatures increase more energy is available for molecules in a liquid to break their bonds and enter a gaseous phase at a faster rate, with highly volatile chemicals often requiring less energy than liquids with lower volatility (Ochiai et al. 2012; Price 2019). Likewise, the general negligible effect of humidity on odorant loss is also generally reported in the literature for artificial dispensers such as polyethylene bulbs (Hall et al. 1997; Klassen et al. 2023; McDonough 1997; Nielsen et al. 2019). However, there are exceptions: Tomaszewska et al. (2005) found that air relative humidity above 80% resulted in a water barrier forming on the surface of aerogel matrix dispensers, thus negatively impacting odour diffusion ability. Additionally, Veršić Bratinčević et al. (2023) state that the evaporation of an odorant from a dispenser depends not just on environmental factors, like temperature or humidity, but on the type of dispenser used as well as the chemical nature of the odorant. The significant effect of humidity as well as the interaction of humidity and temperature on ethyl butyrate does not match with the rest of the results or what the literature indicates as the general trend for dispensers like polyethylene bulbs, making it one of the exceptions. Literature on the effect of humidity on ethyl butyrate mostly involves the field of food storage (López-Carballo et al. 2005; Yoshii et al. 2001). In these cases, high relative humidity decreased the efficacy of the barriers or microencapsulation in preventing ethyl butyrate volatization rather than directly influencing ethyl butyrate volatility (López-Carballo et al. 2005; Yoshii et al. 2001). Additionally, the studies that do test ethyl butyrate as an insect attractant only mention humidity under rearing or experimental conditions and do not check for any effect of humidity on ethyl butyrate release (Coutinho-Abreu et al. 2014; Cruz-López et al. 2006; Guarino et al. 2011; Massa et al. 2008). This makes it difficult to determine a possible cause for ethyl butyrate release being affected by relative humidity in this study.

The GCMS analysis indicates that there was no contamination or secondary compound formation as a result of exposure to environmental factors such as direct sunlight which is known to influence odorant release, profile, and the dispenser itself (Leskey et al. 2005; Nielsen et al. 2019; Torr et al. 1997). We also found that the volume in the bulbs decreased from Day 0 through to Day 7. This result is similar to results obtained by Hammack and Petroski (2004), where relative release rate in micromoles per hour increased as dosage was increased on a cotton dispenser. The decline in release rate as polyethylene dispensers age in the field has also been noted by Bradley et al. (1995) while working on developing a predictive temperature dependent model for pheromone release rates from polyethylene dispensers.

Yellow sticky traps not augmented in any way are known to be effective at monitoring psyllid populations, even at low densities (Hall et al. 2010; Miranda et al. 2018; Monzo et al. 2015). During our study, yellow sticky traps in field cages with a known *T. erytreae* population caught roughly 10–15% of the psyllids released on average. Furthermore, augmentation with semiochemical lures did not significantly increase nor decrease *T. erytreae* catches on the yellow sticky traps under semi-field and field conditions. Considering that the total cost for the chemicals, bulbs, and commercial *D. citri* lures were nearly twice as much as the yellow sticky traps used in the study with no significant difference in *T. erytreae* catch using odorants possibly attractive to both *D. citri* and *T. erytreae* at any level of the experiment, it would currently not be cost-effective to use odorants tested in this study to improve yellow sticky trapping for the two psyllid vectors of HLB. Not even the already available ACP Pherolure, which is effective at improving *D. citri* catch on suitably coloured sticky traps (Sétamou et al. 2018), was effective at improving *T. erytreae* catch on the traps. There is also the potential issue of populations of the same pest species from different continents responding differently to semiochemicals to consider (Borden et al. 1982; Nielsen 2013). Blends of several semiochemicals in specific ratios, tested on local populations or on populations from regions that are likely to be the source of invasions in the case of species not yet present in the country, may need to be explored in the future for improved monitoring and potential tools for citrus psyllids (Borden et al. 1982; Martini et al. 2020; Nielsen 2013). Even then, it would depend on whether the improved efficacy of the lure would outweigh the cost of the blend.

## Conclusion

Yellow sticky traps without any of the odorants tested in this study would be effective for monitoring of the HLB psyllid vectors in South African citrus orchards. None of the high concentration, single semiochemical lures tested under semi-field conditions had a significant improvement effect on yellow sticky trap efficacy. For selected odorants, neither high and low concentrations of the chemicals were effective at improving yellow sticky trap efficacy for *T. erytreae*. Yellow traps have proven to be attractive to both *D. citri* and *T. erytreae* by acting as a standard visual cue for host finding via mimicking the yellow/green colouration of fresh flush growth which both species utilize. The use of odorants and odorant blends with yellow sticky traps should, however, not be completely ruled out. Further work is however required on effective dispensing mechanism and blend ratios. This study provided a baseline for further work in this field. Ethyl butyrate and ocimene can possibly be further explored for *T. erytreae*. This study provided empirical evidence of the release dynamics of a range of semiochemicals from polyethylene bulbs as affected by temperature. Ethyl butyrate was the only semiochemical affected by humidity in a significant manner and the reason remains unclear. It was clear from this study that lures dispensed from polyethylene bulbs do not have uniform release rates, with release rates declining over time.

## Electronic Supplementary Material

Below is the link to the electronic supplementary material.

Supplementary Material 1

## Data Availability

The experimental data that support the findings of this study are publicly available. The data can be found here: 10.25403/UPresearchdata.25028219.
