# Supplementary material for: Addition of Selected Plant-Derived Semiochemicals to Yellow Sticky Traps Does Not Improve Citrus Psyllid Captures
Source: J Chem Ecol. 2024 Apr 3;50(11):701–13. doi: 10.1007/s10886-024-01491-0 (PMC11543742; doi:10.1007/s10886-024-01491-0)
Supplement: Supplementary file 1 — Supplementary Material 1 [file 10886_2024_1491_MOESM1_ESM.docx]

**ADDITION OF SELECTED PLANT-DERIVED SEMIOCHEMICALS TO YELLOW STICKY TRAPS DOES NOT IMPROVE CITRUS PSYLLID CAPTURES**

Journal of Chemical Ecology

PULLOCK, D. A.^1^, KRÜGER, K.^1, 2^, MANRAKHAN, A.^3,4^, YUSUF, A. A.^1^, AND WELDON, C. W.^1,^ *

1. Department of Zoology and Entomology, Forestry and Agricultural Biotechnology Institute (FABI), University of Pretoria, Hatfield 0028 Pretoria, South Africa
2. KWS SAAT SE & Co. KGaA, 37574 Einbeck, Germany
3. Citrus Research International, Mbombela 1200, South Africa.
4. Department of Conservation Ecology and Entomology, Stellenbosch University, Faculty of AgriSciences, Stellenbosch University, Stellenbosch, Private Bag X1, Matieland 7602, South Africa

*Corresponding author: cwweldon@zoology.up.ac.za

**Table S1 Trap layout in a 5×6 mature lemon tree grid per week over 5 weeks. 1=Hexane, 2=Ocimene, 3=Ethyl butyrate, 4=Control, 5=1% Ocimene, and 6=5% Ethyl butyrate. Trap positions per week were randomised using randomizer.org**

| Week 1 | Week 2 | Week 3 | Week 4 | Week 5 |
| --- | --- | --- | --- | --- |
| 2,5,6,3,2  3,4,2,5,6  5,1,4,2,3  1,3,1,1,5  4,6,3,4,2  6,4,5,6,1 | 5,3,4,1,2  1,1,5,3,6  2,6,2,5,4  6,5,3,4,1  3,4,6,2,5  4,2,1,6,3 | 1,3,2,3,4  3,4,6,5,3  4,5,4,6,1  6,2,3,4,6  5,6,1,2,5  2,1,5,1,2 | 4,2,6,1,5  6,4,2,5,2  2,5,3,6,1  1,6,1,4,3  5,3,4,3,6  3,1,5,2,4 | 5,4,6,5,2  2,1,5,3,1  3,6,3,6,3  4,5,4,1,5  6,3,2,2,4  1,2,1,4,6 |

**Table S2 Comparison between baited and unbaited yellow sticky trap psyllid catch after 3 and 7 days using Wilcoxon signed-rank tests**

| **Day 3**  **Odorant** | ***W*** | **P** | **Odorant** | ***W*** | **P** |
| --- | --- | --- | --- | --- | --- |
| Acetic acid | 13 | 0.518 | Limonene | 5 | 0.149 |
| ACP Pherolure | 1.5 | 0.074 | Methyl  salicylate | 17 | 0.944 |
| p-Cymene | 14 | 1 | Myrcene | 10 | 1 |
| Ethyl butyrate | 24 | 0.441 | Ocimene | 22.5 | 0.573 |
| Hexane | 8 | 0.094 | Sabinene | 17 | 0.944 |
| **Day 7**  **Odorant** | ***W*** | **P** | **Odorant** | ***W*** | **P** |
| Acetic acid | 10.5 | 0.321 | Limonene | 6 | 0.396 |
| ACP Pherolure | 7.5 | 0.156 | Methyl  salicylate | 14.5 | 0.673 |
| p-Cymene | 12 | 0.800 | Myrcene | 14 | 0.34 |
| Ethyl butyrate | 18.5 | 0.497 | Ocimene | 18 | 0.551 |
| Hexane | 10.5 | 0.172 | Sabinene | 17 | 0.944 |


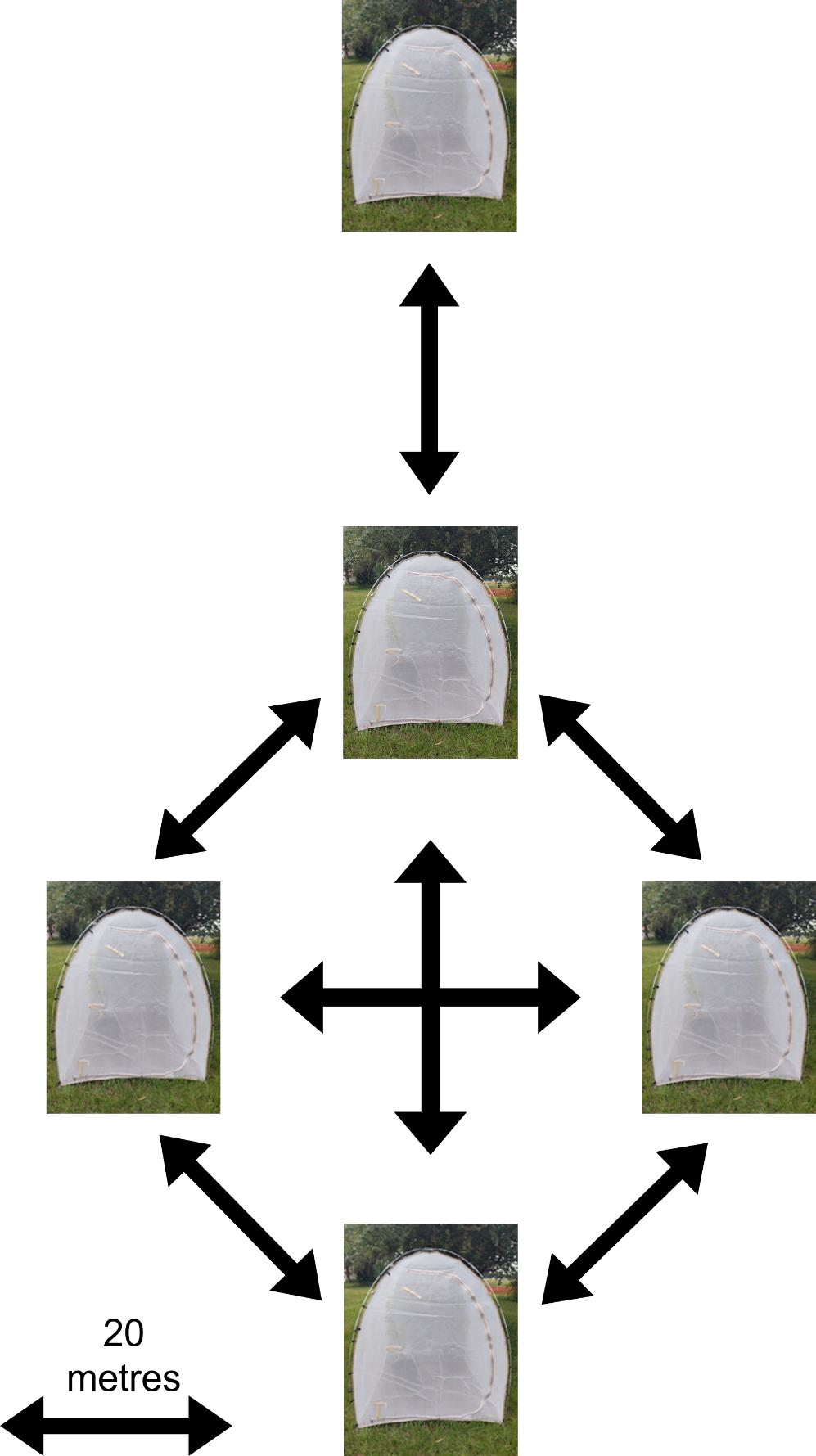


**Fig. S1** Schematic of field cage setup at Innovation Africa campus, University of Pretoria


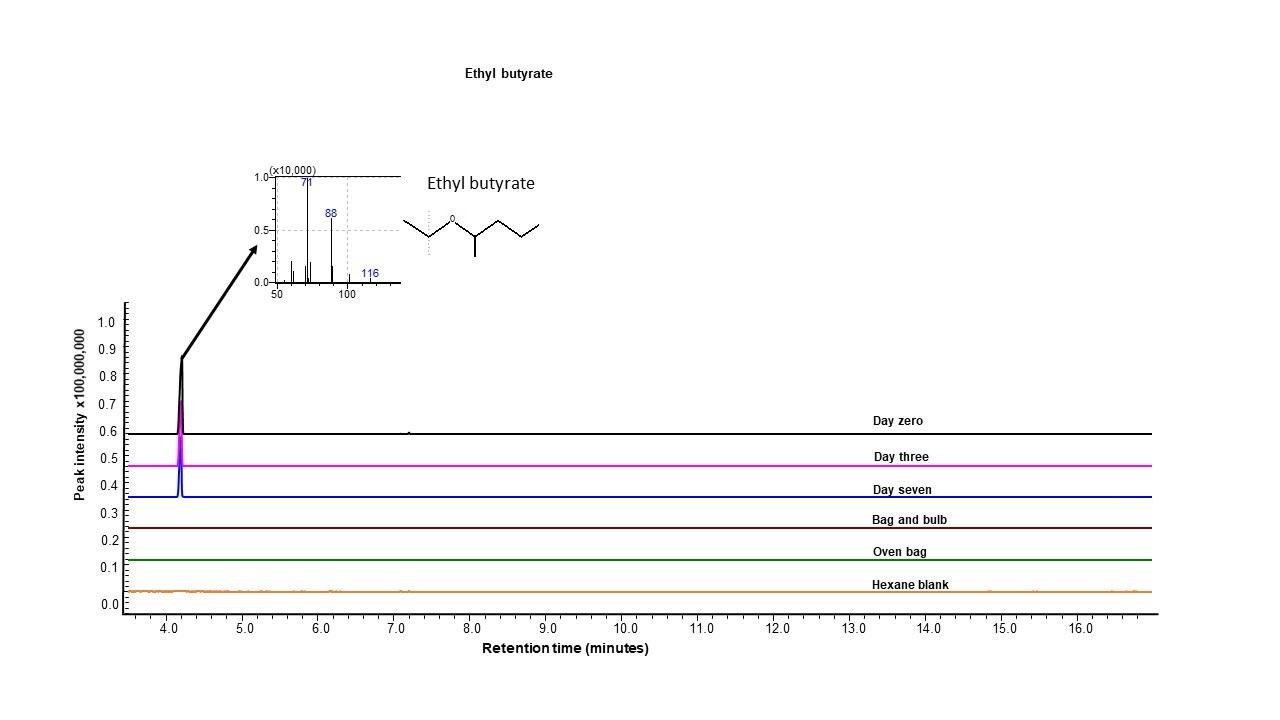

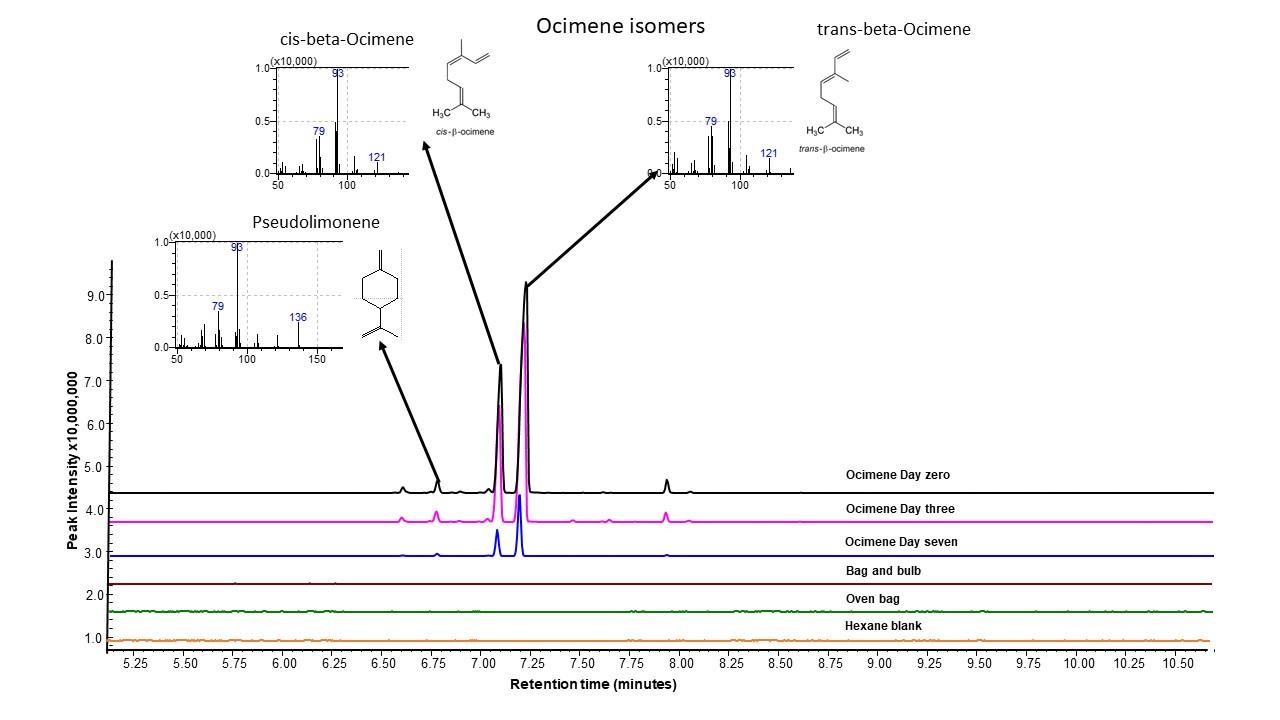


a)

b

)

**Fig. S2** Total ion chromatograms (TICs) depicting the peak intensities of a) Ethyl butyrate, and b) Ocimene isomer mix collected over one hour at Day 0, Day 3, and Day 7. A bag only, bag and bulb, and hexane blank were used to isolate readings from the test odorants


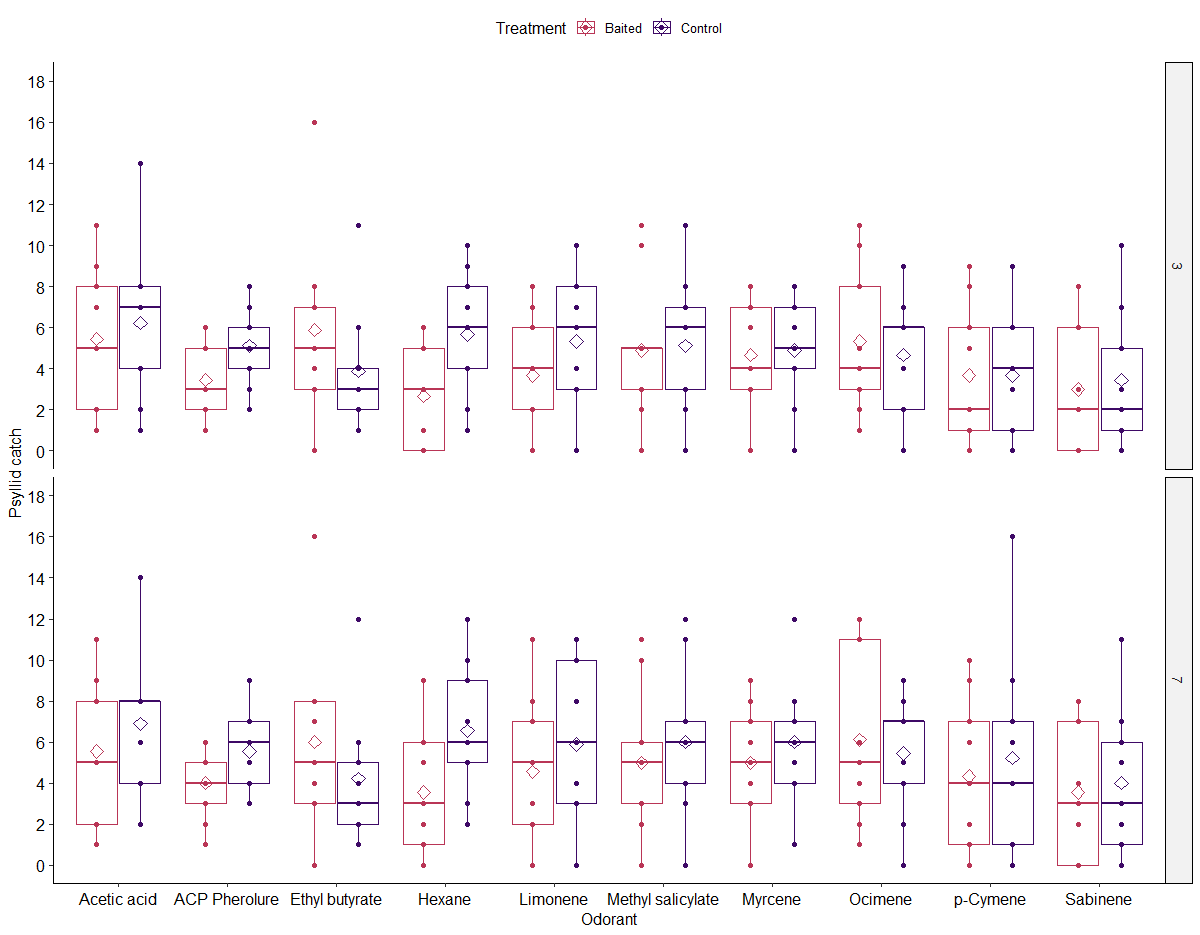


**Fig. S3** Boxplots depicting the psyllid catches on baited and their corresponding unbaited yellow sticky traps after 3 and 7 days. The diamonds indicate mean psyllid catches
